# Single-Incision vs. Conventional Laparoscopic Surgery for Colorectal Cancer: An Update of a Systematic Review and Meta-Analysis

**DOI:** 10.3389/fsurg.2021.704986

**Published:** 2021-08-23

**Authors:** Ye Yuan, Jianing Jian, Hailiang Jing, Ran Yan, Fengming You, Xi Fu, Linke Du, Wenyuan Li

**Affiliations:** ^1^Hospital of Chengdu University of Traditional Chinese Medicine, Chengdu, China; ^2^Sichuan Evidence-Based Medicine Center of Traditional Chinese Medicine, Chengdu, China; ^3^TCM Regulating Metabolic Diseases Key Laboratory of Sichuan Province, Hospital of Chengdu University of Traditional Chinese Medicine, Chengdu, China

**Keywords:** single-incision laparoscopic surgery, conventional laparoscopic surgery, colorectal cancer, randomized controlled trials, meta-analysis, systematic review

## Abstract

**Background:** Although the advantages of single-incision laparoscopic surgery have been reported in several meta-analyses, the low quality of studies included in the meta-analyses limits the reliability of such a conclusion. In recent years, the number of randomized controlled trials on the efficacy of SILS in colorectal cancer has been on the rise. This update systematic review and meta-analysis of RCTs aims to compare efficacy and safety of SILS and CLS in the patients with colorectal cancer.

**Methods:** Relevant data was searched on the CNKI, Wanfang, VIP, Sinomed, PubMed, Embase, and Cochrane CENTRAL databases from inception until February 5th, 2021. All RCTs comparing SILS and CLS were included. The main outcomes were 30 days of mortality, postoperative complications, intraoperative complications, whereas secondary outcomes were the number of lymph nodes removed, duration of hospital stay, intraoperative blood loss, abdominal incision length, reoperation, readmission, conversion to laparotomy, operation time and anastomotic leakage.

**Results:** A total of 10 RCTs were included, involving 1,133 participants. The quality of the included studies was generally high. No significant difference was found between SILS and CLS in the 30 days mortality rate. The results showed that SILS group had a lower rate of postoperative complications (RR = 0.67, 95% CI: 0.49–0.92), higher rate of intraoperative complications (RR = 2.26, 95%CI: 1.00–5.10), shorter length of abdominal incision (MD = −2.01, 95% CI:−2.42–1.61) (cm), longer operation time (MD = 11.90, 95% CI: 5.37–18.43) (minutes), shorter hospital stay (MD = −1.12, 95% CI: −1.89–0.34) (days) compared with CLS group. However, intraoperative blood loss (MD = −8.23, 95% CI: −16.75–0.29) (mL), number of lymph nodes removed (MD = −0.17, 95% CI: −0.79–0.45), conversion to laparotomy (RR=1.31, 95% CI: 0.48–3.60), reoperation (RR = 1.00, 95% CI: 0.30–3.33) and readmission (RR =1.15, 95% CI: 0.12–10.83) and anastomotic leakage were not significantly different between the two groups.

**Conclusion:** These results indicate that SILS did not has a comprehensive and obvious advantage over the CLS. Surgeons and patients should carefully weigh the pros and cons of the two surgical procedures. Further RCTs are needed to prove long-term outcomes of SILS in colorectal cancer.

## Introduction

Colorectal cancer (CRC) is the third most commonly diagnosed cancer in the world (1). Surgical resection is the only curative treatment for CRC (2, 3). In the past 60 years, general surgery has radically changed to minimally invasive surgery techniques to enhance the recovery rate (4). Since the first laparoscopic surgery was performed over 100 years ago by Jacobaeus (5), minimally invasive surgery has continued to play an important role as an alternative to traditional open surgery. Laparoscopic surgery demonstrates faster functional recovery rates, fewer postoperative complications, shorter length of the incision, and shorter hospital stay when compared with open surgery. Therefore, laparoscopic surgery is gaining acceptance as an alternative treatment option for colorectal cancer (6).

Recent innovations in surgical techniques such as robot-assisted laparoscopic surgery (RALS), single-incision laparoscopic surgery (SILS), and natural orifice transluminal endoscopic surgery (NOTES), etc, have greatly benefited patients with colorectal cancer (7–9). NOTES has gained significant attention because it offers the possibility of “scarless” surgery (10). However, the clinical application of NOTEs has been limited due to several unresolved problems such as limitations of surgical techniques and equipment, unregulated insufflations and narrow working angles, etc., (11). SILS is regarded as an alternative surgical technique for NOTES and the next major advance in minimally invasive surgical methods for colorectal cancer (12). In SILS, the surgeon operates through a single incision, and it is generally considered to have the following advantages, less postoperative pain, better cosmetic effect, less postoperative complications, less intraoperative blood loss, shorter hospital stay and shorter length of skin incision, etc., when compared with conventional laparoscopic surgery (CLS) (13, 14). However, SILS presents some new technical challenges compared with CLS (15, 16), such as (1) the limited number of working instruments which makes it difficult to achieve correct exposure and the necessary traction to tissues; (2) Limited external working space: multiple instruments and laparoscopies required for a procedure compete for the same space at the entry port, leading to external hand collisions and difficulty in internal manipulation of the instrument tip compared with CLS; (3) difficult to maintain pneumoperitoneum; (4) Requirement of training and adjustment. The skills required for SILS differ from those required for CLS, including laparoscopic surgeons' experienced, and skills. Besides, colorectal surgery magnifies all the challenges of SILS. Unlike laparoscopic cholecystectomy or appendectomy, which only involves surgery in one abdominal quadrant, single-incision laparoscopic colectomy requires operating in different abdominal quadrants. However, there is no clinical evidence confirming the feasibility and safety of SILS for colorectal cancer.

The number of studies on SILS for colorectal cancer has increased seven-fold between 2010 and 2021. Randomized controlled trials (RCTs) comparing single-incision vs. conventional laparoscopic surgery for colorectal cancer are reported. Consequently, this systematic review and meta-analysis aims to compare efficacy and safety of SILS and CLS in the patients with colorectal cancer. The study included only randomized controlled trials.

## Materials and Methods

### Study Design

This study followed the Preferred Reporting Items for Systematic Reviews and Meta-Analyses guidelines (17) (PRISMA) and was registered in PROSPERO, Registration number: CRD42021232237.

### Search Strategy

PubMed, Embase, Cochrane CENTRAL, CNKI, Sinomed, and Wan Fang databases were searched through February 5th, 2021 by two independent researchers. The Chinese search terms used were “jiechangai” “zhichangai” “jiezhichangai” “dachangai” “changzhongliu” “fuqiangjing” “dankong” “danqiekou”. The English search terms used were “colon cancer” “colorectal cancer” “rectal cancer” “sigmoid cancer” “single-incision laparoscopic surgery” “conventional laparoscopic surgery” and “randomized controlled trials”. Different search strategies were adapted for each database (Table 1). References of included studies were also examined to find relevant studies.

**Table 1:** PubMed search strategy of single-incision vs. conventional laparoscopic surgery for colorectal cancer study.

**Number**	**Search terms**
#1	“colon neoplasm” [MeSH] OR “colon carcinoma” [Title/Abstract] OR “colon cancer” [Title/Abstract] OR “colon tumor” [Title/Abstract] OR “colonic neoplasm” [Title/Abstract] OR “colonic carcinoma” [Title/Abstract] OR “colonic cancer” [Title/Abstract] OR “colonic tumor” [Title/Abstract] OR “colorectal neoplasm” [Title/Abstract] OR “colorectal carcinoma” [Title/Abstract] OR “colorectal cancer” [Title/Abstract] OR “colorectal tumor” [Title/Abstract]
#2	“rectal neoplasm” [MeSH] OR “rectal carcinoma” [Title/Abstract] OR “rectal cancer” [Title/Abstract] OR “rectal tumor” [Title/Abstract]
#3	“sigmoid neoplasm” [MeSH] OR “sigmoid carcinoma” [Title/Abstract] OR “sigmoid cancer” [Title/Abstract] OR “sigmoid tumor” [Title/Abstract]
#4	“single-incision” [Title/Abstract] OR “single-site” [Title/Abstract] OR “single-port” [Title/Abstract]
#5	“laparoscopy” [Title/Abstract] OR “surgery” [Title/Abstract] OR “laparoscopic” [Title/Abstract] OR “laparoscopic surgery” [Title/Abstract]
#6	#1 OR #2 OR #3
#7	#4 AND #5 AND #6

### Inclusion and Exclusion Criteria

Inclusion criteria: (1) Patients were diagnosed with colon cancer or rectal cancer; (2) Patients not less than 18 years old; (3) UICC stage 0-III or Dukes stage A-C; (4) The intervention in the experimental group was single-incision laparoscopic surgery, and conventional laparoscopic surgery used in the control group; (5) Main outcomes: 30 days of mortality, postoperative complications, intraoperative complications; (6) Secondary outcomes: number of lymph nodes removed, hospital stay, intraoperative blood loss, abdominal incision length, reoperation, readmission, conversion to laparotomy and operation time; (7) If there were multiple reports that came from the same study, the latest report were included; (8) Randomized controlled trials; (9) Type of surgery is resection. Exclusion criteria**: (**1) Patients with other malignancies diagnosed within the past 5 years; (2) Pregnant or lactating patients; (3) American Society of Anesthesiologists (ASA) class>III; (4) Emergency cancer surgery due to perforation or obstruction; (5) No outcomes available in the studies.

### Data Extraction and Management

A predefined data extraction table was used by two independent researchers to extract relevant information, including (publication year, author, title), demographic information (the number of participants in the treatment group and the control group, gender, average age, diagnosis method, inclusion and exclusion criteria), intervention feature information (explanation of the surgical procedure) and methodological elements (random sequence generation, allocation concealment, blinding of participants and personnel, blinding of outcome assessment, incomplete outcome data, selective reporting, and other bias). Any disagreements on information extracted by the two researchers were resolved by a third researcher after discussion with the two researchers.

### Assessment of Risk of Bias

A 7-point Jadad scale (18) was used to assess the quality of the identified studies and includes four assessment items: randomization (2 points), allocation concealment (2 points), blinding methods (2 points), and withdrawals (1 point). A score of 0 to 7 was assigned, and higher scores indicated higher quality. Any study scoring at least 4 was considered to have high methodology quality, and disagreements between the two researchers were resolved by a third researcher who would make the final decision.

### Statistical Analysis

RevMan 5.3 and R 4.02 were used to perform all the statistical analyses. Similar populations, interventions, and outcomes were combined in the study. For dichotomous data and continuous variables, the inverse variance method and the Mantel-Haenszel method were used. Otherwise, relative risk (RR) or mean difference (MD) were used for both types of data to compare the treatment results with a 95% confidence interval (95% CI). We assessed heterogeneity by χ^2^ test and I^2^ statistics. I^2^≥0.1 and P ≤ 50% indicated acceptable heterogeneity, using a fixed-effect model to analyze effect quantities. Conversely, *I*^2^ < 0.1 or *P* > 50% suggests significant heterogeneity, using a random-effect model to analyze effect quantities. Sensitivity analysis was used to identify clinical heterogeneity, after excluding studies with obvious clinical heterogeneity, the fixed-effects model was used to calculate the combined effect or qualitative description. Subgroup analysis was performed according to the cancer type and previous history of major abdominal surgery in patients in both groups. The funnel plot and egger's test were used to test for publication bias of main outcomes.

### Quality of Evidence Assessment

Use GRADE profiler 3.6 was used to evaluate the quality of the study results. The evaluation criteria included five aspects: risk bias, inconsistency, indirectness, imprecision, and publication bias. Finally, the quality of evidence was divided into four levels: high quality, medium quality, low quality, and very low quality.

## Results

### Study Characteristics

**Figure 1:**
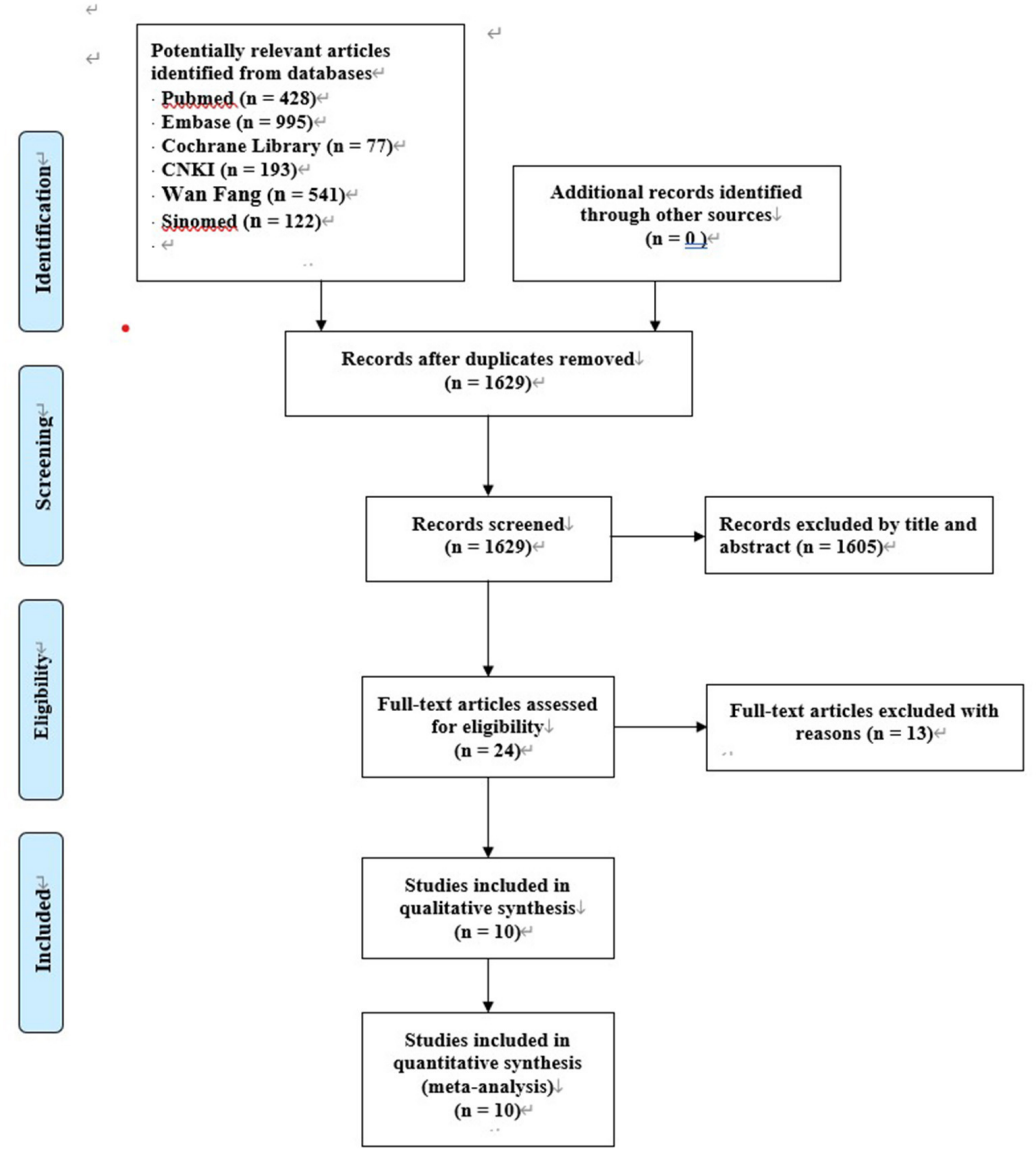
PRISMA diagram of literature searching and screening process.

A total of 2,356 entries were obtained by searching the Chinese and English databases and 1,629 entries were obtained after excluding duplicate entries. Further, 1,605 entries were excluded by reading the titles and abstracts. The full text of the remaining 24 articles was downloaded and 11 articles were included in this systematic review and meta-analysis after reading the entire article. Finally, ten RCTs with 1,133 participants were included in this study, 566 participants were enrolled in the SILS group and 567 participants were enrolled in the CLS group. All included RCTs were published between 2012 and 2020. Figure 1 shows this study's literature searching and screening process and Table 2 presents a summary of the included studies.

**Table 2:** Main characteristics of the selected studies.

**Reference**	**Country/Area**	**Year**	**Sample size**		**Age**		**Gender (male/female)**		**BMI**		**Tumor diameter(cm)**		**Disease**	**Outcome**	**Jadad score**
			**SILS**	**CLS**	**SILS**	**CLS**	**SILS**	**CLS**	**SILS**	**CLS**	**SILS**	**CLS**			
Wu et al. (19)^*^	China	2020	49	49	61.89 ± 7.50	62.04 ± 7.2	29/20	31/18	23.11 ± 2.69	23.05 ± 2.81	3.95 ± 0.40	3.88 ± 0.49	Colorectal cancer	defghi	4
Bulut et al. (20)^*^	Denmark	2014	20	20	69 (50–86)	73 (50–84)	8/12	8/12	24 (16–32)	24 (19–29)	/	/	Rectal cancer	bgjk	4
Huscher et al. (21)^*^	Italy	2012	16	16	70 ± 11	70 ± 13	6/10	9/7	/	/	/	/	Colon cancer	abcfhi	5
Kang et al. (22)^#^	Korea	2016	31	31	63.2–11.4	63.2–11.4	19/12	16/15	24.0 ± 3.0	24.5 ± 3.0	5.3 ± 2.0	4.5 ± 2.9	Colon cancer	abcdfghik	5
Lee et al. (23)^#^	Korea	2020	179	180	63.4 (34–84)	62.6 (28–85)	97/82	99/81	24.3 (17.0–32.0)	24.3 (18.0–35.0)	3.7 (0–9.0)	3.5 (0–9.5)	Colon cancer	bcgk	4
Poon et al. (24)^#^	Hong Kong	2012	25	25	67 (37–83)	67 (57–81)	14/11	18/7	23.2 (16.9–28.8)	23.6 (16.5–28.2)	3.5 (1–7)	4 (1–7)	Colon cancer	b	4
Chen et al. (25)^*^	China	2017	43	43	54.39 ± 11.66	54.87 ± 10.98	27/16	25/18	22.01 ± 2.10	21.87 ± 2.02	3.31 ± 0.31	3.40 ± 0.45	Colorectal cancer	bdefhi	2
Watanabe et al. (26)^*^	Japan	2016	100	100	66.7	66.6	56/44	56/44	23.1	23.2	2.75	2.77	Colorectal cancer	abgj	4
Wang et al. (27)^*^	China	2018	60	60	55.24 ± 7.88	55.86 ± 7.28	32/28	36/24	26.02 ± 2.84	25.38 ± 2.64	3.62 ± 1.48	3.58 ± 1.65	Colorectal cancer	bdefhi	4
Xu (28)	China	2019	43	43	47.92 ± 5.58	47.89 ± 5.61	27/16	25/18	/	/	/	/	Rectal cancer	bdefhi	4

* *: Major abdominal surgery history*;

# *: No major abdominal surgery history; a: 30 days of mortality; b: Postoperative complications; c: Intraoperative complications; d: Length of abdominal incision; e: Intraoperative blood loss; f: Number of lymph nodes removed; g: Conversion to laparotomy; h: Operation time; i: Hospital stay; j: Reoperation; k: Readmission; SILS: single-incision laparoscopic surgery; CLS: conventional laparoscopic surgery*.

### Quality Assessment of Included Studies

Only one single study (25) did not mention random sequence generation and allocation concealment, while nine studies (19–24, 26–28) indicated the use of random number tables or other random allocation schemes. The double blind method was not adopted in any of the studies. All studies indicated the reasons for and numbers of withdrawals. The quality of included studies was considered high (Table 2).

### Meta-Analysis Results

#### Main Outcomes

##### The Thirty-Day Mortality

Three studies (21, 22, 26), with 294 patients showed no significant difference between SILS and CLS in 30-day mortality. No deaths were reported in two studies within 30 days (21, 26). Kang et al. (22) reported the death of 1 SILS patient within 30 days.

##### Postoperative Complications

**Figure 2:**
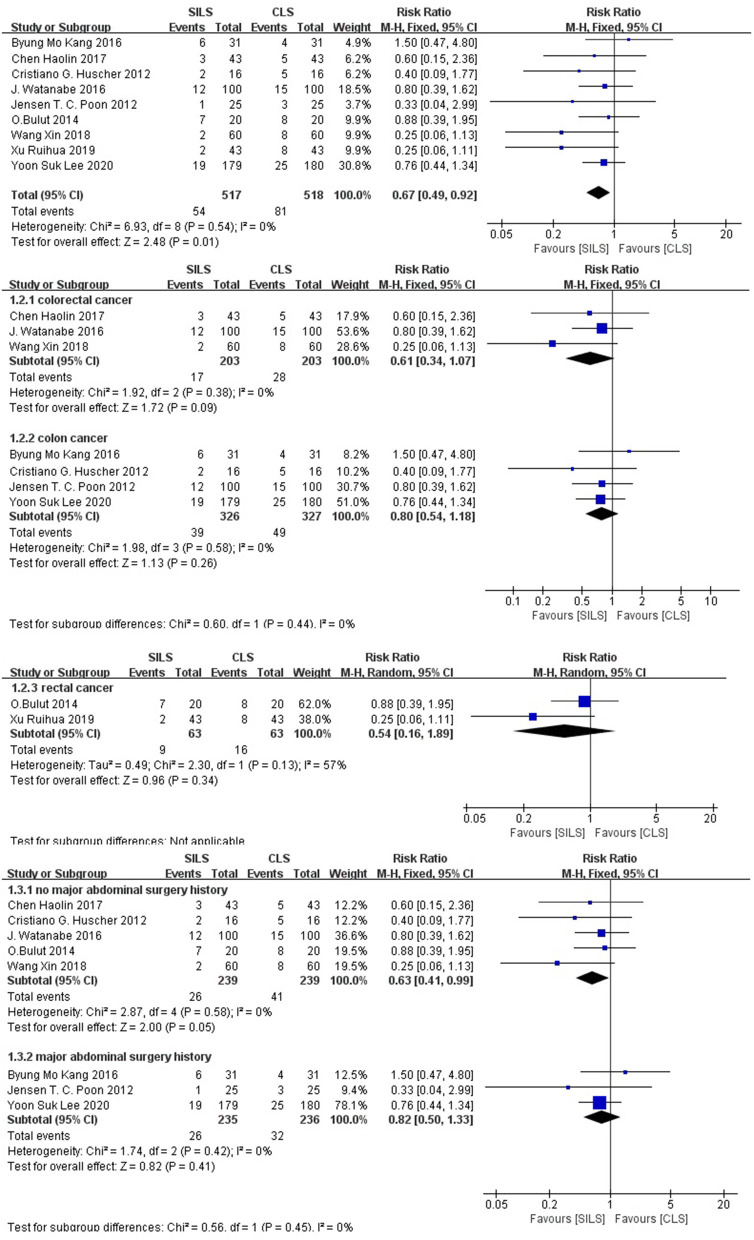
Meta-analysis of postoperative complications. SILS, single-incision laparoscopic surgery; CLS, conventional laparoscopic surgery.

A total of nine studies (20–28) with 1,035 patients reported a high rate of postoperative complications. Heterogeneity test: *P* = 0.54, *I*^2^ = 0, showed no heterogeneity. Fixed-effect model was applied in the analyses. The SILS group showed lower rate of postoperative complications compared with the CLS group [RR = 0.67, 95% CI: 0.49–0.92, *P* = 0.01] (Figure 2).

Subgroup analysis: The patients were divided into three subgroups according to the cancer type. Colorectal cancer: The effects of three studies (25–27) including 406 patients were combined to perform meta-analysis. Heterogeneity test: *P* = 0.38, *I*^2^ = 0, showed no heterogeneity. The fixed-effect model was applied in the analyses. Meta-analysis results [RR = 0.61, 95% CI: 0.34–1.07, *P* = 0.09], showed no statistical significance (Figure 2) Colon cancer: The effects of four studies (21–24), including 653 patients were combined to perform meta-analysis. Heterogeneity test: *P* = 0.58, *I*^2^ = 0, showed no heterogeneity. The fixed-effect model was applied in the analyses. Meta-analysis results [RR = 0.80, 95% CI: 0.54–1.18, P = 0.26], showed no statistical significance (Figure 2) Rectal cancer: The effects of two studies (20, 28), including 126 patients were combined to perform meta-analysis. Heterogeneity test: *P* = 0.13, *I*^2^ = 57%, showed substantial heterogeneity. Random-effect model was applied in the analyses. Meta-analysis result [RR = 0.54, 95% CI: 0.16–1.89, *P* = 0.34], showed no statistical significance (Figure 2).

The patients were divided into two subgroups according to their previous history of major abdominal surgery. No major abdominal surgery history was reported: The effects in five studies (20, 21, 25–27), including 478 patients were combined to perform the meta-analysis. Heterogeneity test: *P* = 0.58, *I*^2^ = 0, showed no heterogeneity. The fixed-effect model was applied in the analyses. Meta-analysis result [RR = 0.63, 95% CI: 0.41–0.99, *P* = 0.05] in the SILS group showed lower rates of postoperative complications than the CLS group. Major abdominal surgery history: The effects in three studies (22–24), including 471 patients were combined to perform the meta-analysis. Heterogeneity test: *P* = 0.42, *I*^2^ = 0, showed no heterogeneity. The fixed-effect model was applied in the analyses. Meta-analysis result [RR = 0.82, 95% CI: 0.50–1.33, *P* = 0.41] showed no statistical significance (Figure 2).

##### Intraoperative Complications

**Figure 3:**
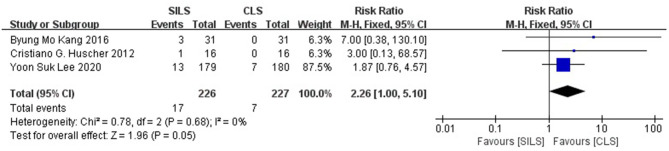
Meta-analysis of intraoperative complications. SILS, single-incision laparoscopic surgery; CLS, conventional laparoscopic surgery.

Three studies (21–23) with a total of 453 patients reported the rate of intraoperative complications. Heterogeneity test: *P* = 0.68, *I*^2^ = 0, showed no heterogeneity. The fixed-effect model was applied in the analyses. The SILS group showed higher rate of intraoperative complications compared with the CLS group [RR = 2.26, 95% CI: 1.00–5.10, *P* = 0.05] (Figure 3).

#### Secondary Outcomes

##### Anastomotic Leakage

Anastomotic leakage may be a postoperative complication of interest to surgeons. In the included studies, the incidence of anastomotic leakage was low. We did not perform quantitative synthesis, but used a qualitative description of the outcome. Eight studies (20–26, 28),with 915 patients showed no significant difference between SILS and CLS in anastomotic leakage. We found that nine patients with anastomotic leakage were found in SILS group with 457 patients and 11 patients with anastomotic leakage were found in CLS group with 458 patients. No anastomotic leakages were reported in three studies (22, 24, 28). Bulut et al. (20) reported that there were four patients with anastomotic leakage in SILS group and CLS group separately. Huscher et al. (21) reported that no patient with anastomotic leakage was found in SILS group and one patient with anastomotic leakage was found in CLS group. Lee et al. (23) reported that two patients with anastomotic leakage were found in SILS group and one patient with anastomotic leakage was found in CLS group. Chen et al. (25) reported that there was one patient with anastomotic leakage in SILS group and CLS group separately. Watanabe et al. (26) reported that two patients with anastomotic leakage were found in SILS group and four patients with anastomotic leakage were found in CLS group.

##### Length of Abdominal Incision (cm)

**Figure 4:**
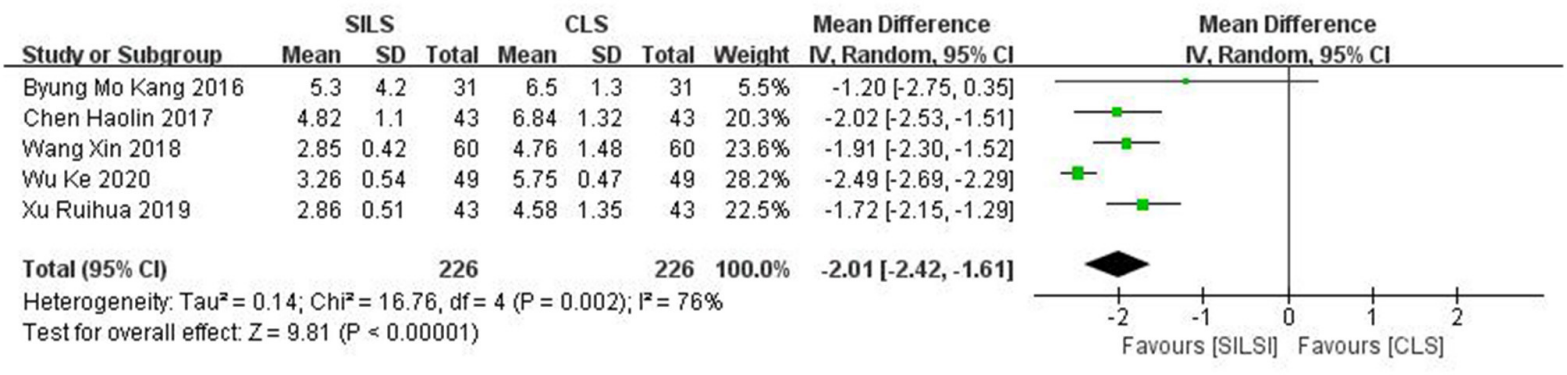
Meta-analysis of length of abdominal incision. SILS, single-incision laparoscopic surgery; CLS, conventional laparoscopic surgery.

Five studies (19, 22, 25, 27, 28) with a total of 452 patients reported the length of abdominal incision. Heterogeneity test: *P* = 0.002, *I*^2^ = 76%, showed high heterogeneity. The random-effect model was applied in the analyses. The SILS group showed shorter length of abdominal incision compared with the CLS group [MD = −2.01, 95% CI: −2.42 to −1.61, *P* < 0.00001] (Figure 4).

##### Intraoperative Blood Loss (mL)

**Figure 5:**
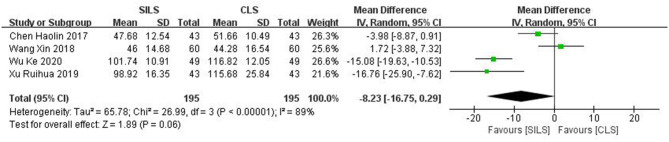
Meta-analysis of intraoperative blood loss. SILS, single-incision laparoscopic surgery; CLS, conventional laparoscopic surgery.

Four studies (19, 25, 27, 28) with a total of 390 patients reported intraoperative blood loss. Heterogeneity test: *P* < 0.00001, *I*^2^ = 89%, showed high heterogeneity. The random-effect model was applied in the analyses. Meta-analysis result [MD = −8.23, 95% CI: −16.75–0.29, *P* = 0.06] showed no statistical significance (Figure 5).

##### Number of Lymph Nodes Removed

**Figure 6:**
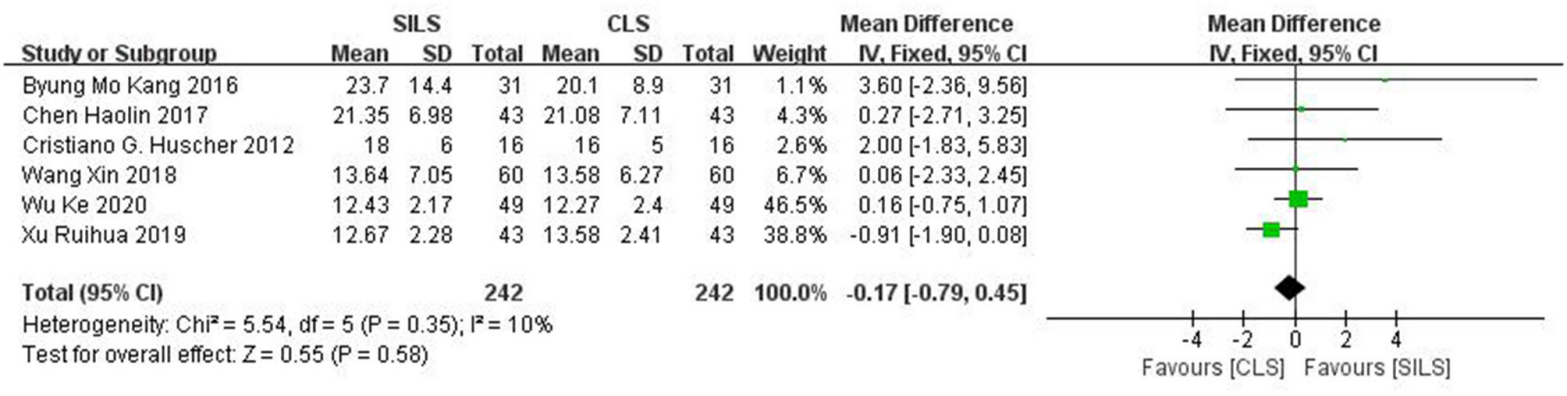
Meta-analysis of number of lymph nodes removed. SILS, single-incision laparoscopic surgery; CLS, conventional laparoscopic surgery.

Six studies (19, 21, 22, 25, 27, 28) with a total of 484 patients reported the number of lymph nodes removed. Heterogeneity test: *P* = 0.35, *I*^2^ = 10%, showed low heterogeneity. The fixed-effect model was applied in the analyses. Meta-analysis result [MD = −0.17, 95%CI: −0.79–0.45, *P* = 0.58] showed no statistical significance (Figure 6).

##### Conversion to Laparotomy

**Figure 7:**
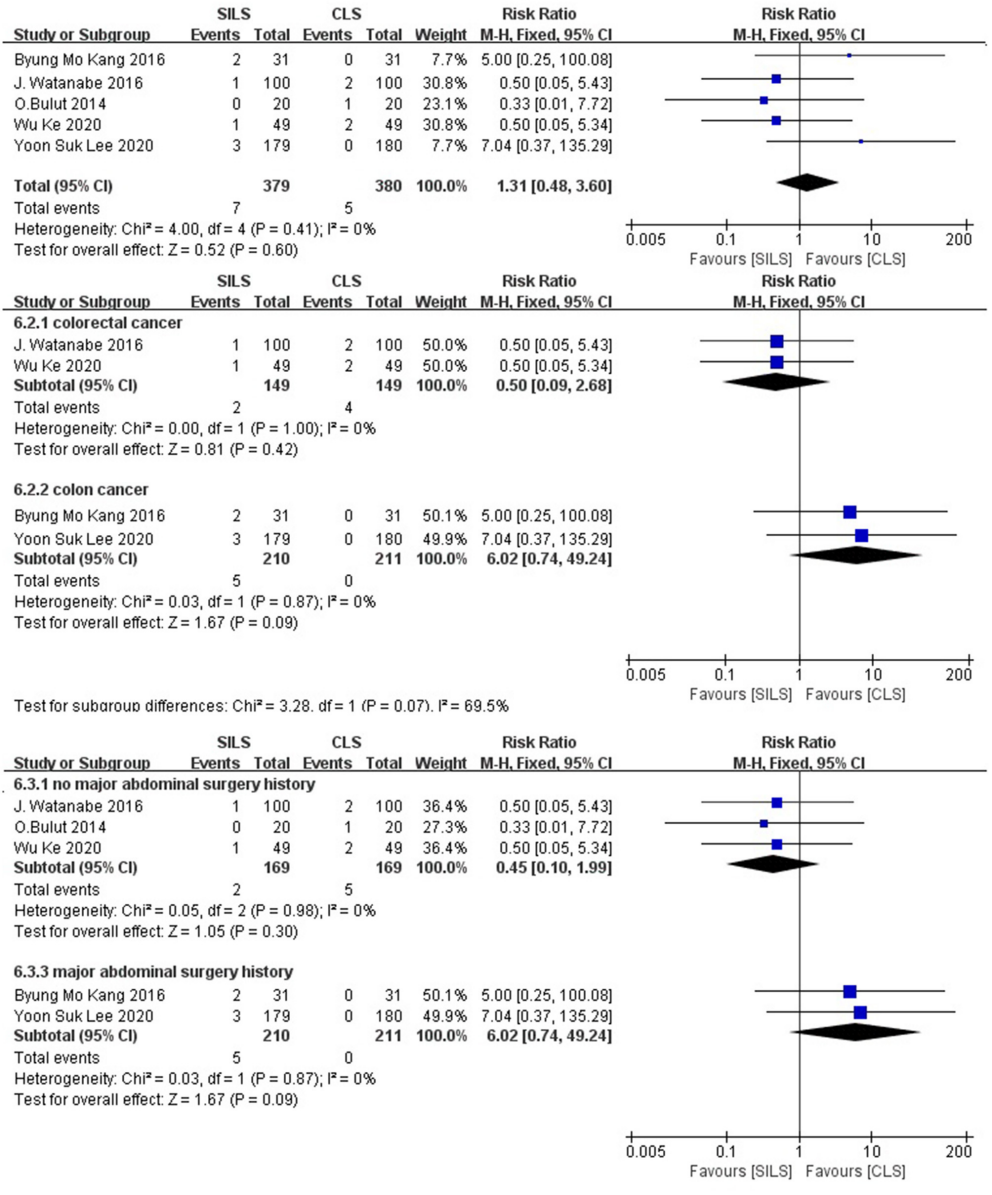
Meta-analysis of conversion to laparotomy. SILS, single-incision laparoscopic surgery; CLS, conventional laparoscopic surgery.

Five studies (19, 20, 22, 23, 26) with a total of 759 patients reported the rate of conversion to laparotomy. Heterogeneity test: *P* = 0.41, *I*^2^ = 0, showed no heterogeneity. The fixed-effect model was applied in the analyses. Meta-analysis result [RR = 1.31, 95% CI: 0.48–3.60, *P* = 0.60] showed no statistical significance (Figure 7).

Subgroup analysis: The patients were divided into two subgroups according to the cancer type. Colorectal cancer: Two studies (19, 26) with a total of 298 patients reported the rate of conversion to laparotomy. Heterogeneity test: *P* = 1.00, *I*^2^ = 0, showed no heterogeneity. The fixed-effect model was applied in the analyses. Meta-analysis results [RR = 0.5, 95% CI: 0.09–2.68, *P* = 0.42] showed no statistical significance. Colon cancer: Two studies (22, 23) with a total of 421 patients reported the operation time. Heterogeneity test: *P* = 0.87, *I*^2^ = 0, showed no heterogeneity. The fixed-effect model was applied in the analyses. Meta-analysis results [RR = 6.02, 95% CI: 0.74–49.24, *P* = 0.09] showed no statistical significance (Figure 7).

The patients were divided into two subgroups according to their previous history of major abdominal surgery. No major abdominal surgery history: The effects of three studies (19, 20, 26), with a total of 338 patients were combined to perform the meta-analysis. Heterogeneity test: *P* = 0.98, *I*^2^ = 0, showed no heterogeneity. The fixed-effect model was applied in the analyses. Meta-analysis results [RR = 0.45, 95% CI: 0.10–1.99, *P* = 0.30], showed no statistical significance. Major abdominal surgery history: The effects of two studies (22, 23), with a total of 421 patients were combined to perform the meta-analysis. Heterogeneity test: *P* = 0.87, *I*^2^ = 0, showed no heterogeneity. The fixed-effect model was applied in the analyses, showed no statistical significance (Figure 7).

##### Operation Time (Minutes)

**Figure 8:**
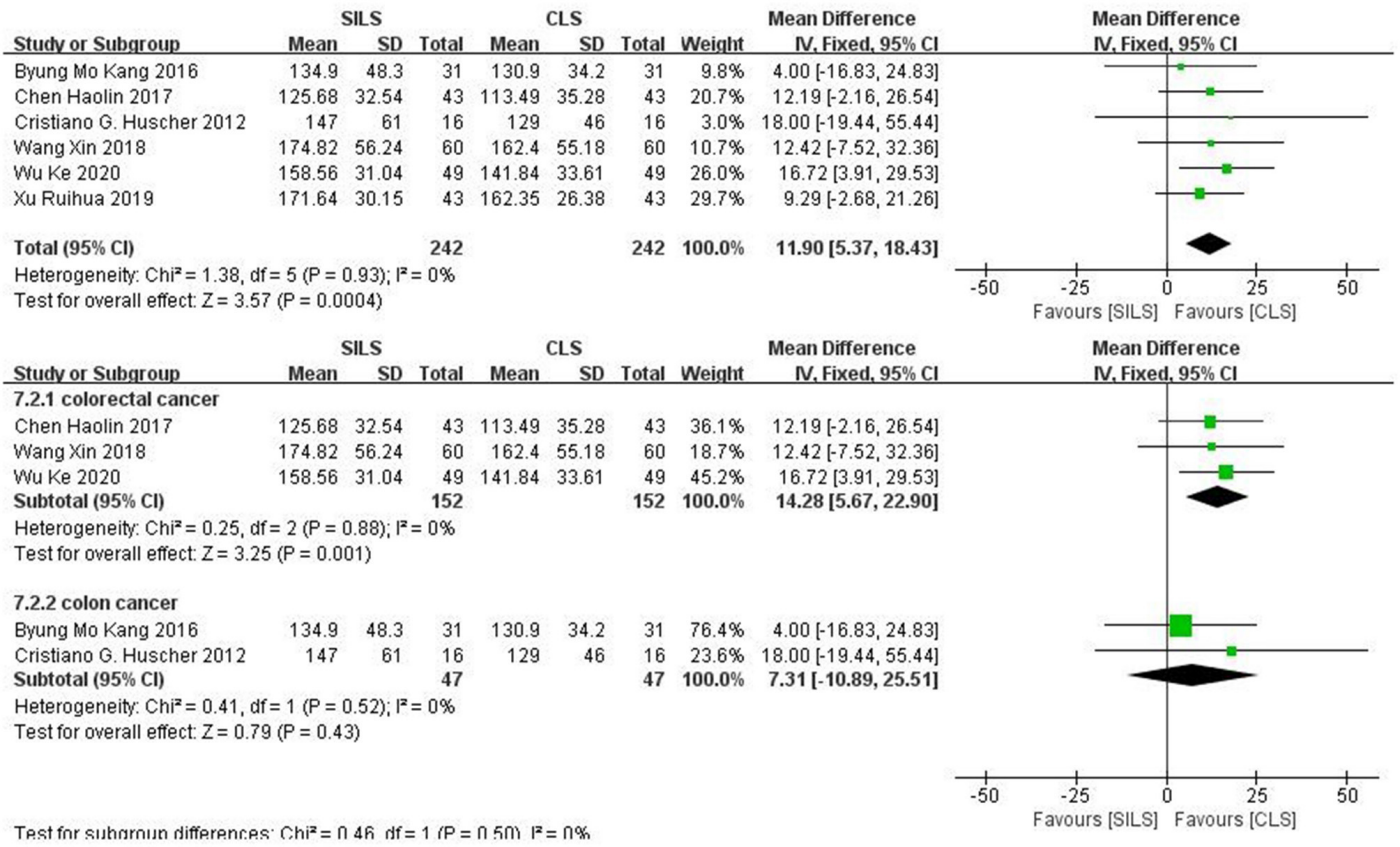
Meta-analysis of operation time. SILS, single-incision laparoscopic surgery; CLS, conventional laparoscopic surgery.

Six studies (19, 21, 22, 25, 27, 28) with a total of 484 patients reported the operation time. Heterogeneity test: *P* = 0.93, *I*^2^ = 0, showed no heterogeneity. The fixed-effect model was applied in the analyses. The SILS group showed longer operation time compared with the CLS group [MD = 11.9, 95% CI: 5.37–18.43, *P* = 0.0004] (Figure 8).

Subgroup analysis: The patients were divided into two subgroups according to the cancer type. Colorectal cancer: Three studies (19, 25, 27) including 304 patients reported the operation time. Heterogeneity test: *P* = 0.88, *I*^2^ = 0, showed no heterogeneity. The fixed-effect model was applied in the analyses. The SILS group showed longer operation time compared with the CLS group [MD = 14.28, 95% CI: 5.67–22.9, *P* = 0.001]. Colon cancer: Two studies (21, 22) including 94 patients reported the operation time. Heterogeneity test: *P* = 0.52, *I*^2^ = 0, showed no heterogeneity. The fixed-effect model was applied in the analyses. Meta-analysis result [MD = 7.31, 95% CI: −10.89–25.51, *P* = 0.43] showed no statistical significance (Figure 8).

##### Length of Hospital Stay (Days)

**Figure 9:**
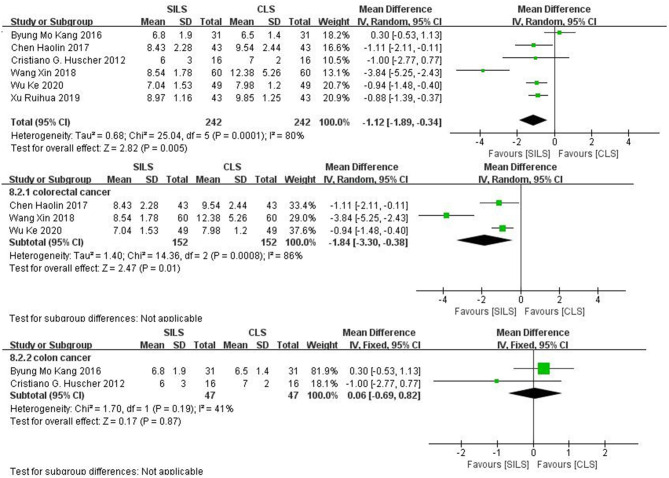
Meta-analysis of length of hospital stay. SILS, single-incision laparoscopic surgery; CLS, conventional laparoscopic surgery.

Six studies (19, 21, 22, 25, 27, 28) with a total of 484 patients reported the length of hospital stay. Heterogeneity test: *P* = 0.0001, *I*^2^ = 80%, showed high heterogeneity. The random-effect model was applied in the analyses. The SILS group showed shorter hospital stay compared with the CLS group [MD = −1.12, 95%CI: −1.89 to −0.34, *P* = 0.005] (Figure 9).

Subgroup analysis: The patients were divided into two subgroups according to the cancer type. Colorectal cancer: Three studies (19, 25, 27) with a total of 304 patients reported the length of hospital stay. Heterogeneity test: *P* = 0.0008, *I*^2^ = 86%, showed high heterogeneity. The random-effect model was applied in the analyses. SILS group shows shorter hospital stay compared with the CLS group [MD = −1.84, 95%CI: −3.30 to −0.38, P = 0.01] (Figure 9). Colon cancer: Two studies (21, 22) with a total of 94 patients reported the length of hospital stay. Heterogeneity test: *P* = 0.19, *I*^2^ = 41%, showed moderate heterogeneity. The fixed-effect model was applied in the analyses. Meta-analysis results [MD = 0.06, 95%CI: −0.69–0.82, *P* = 0.87] showed no statistical significance (Figure 9).

##### Reoperation

**Figure 10:**
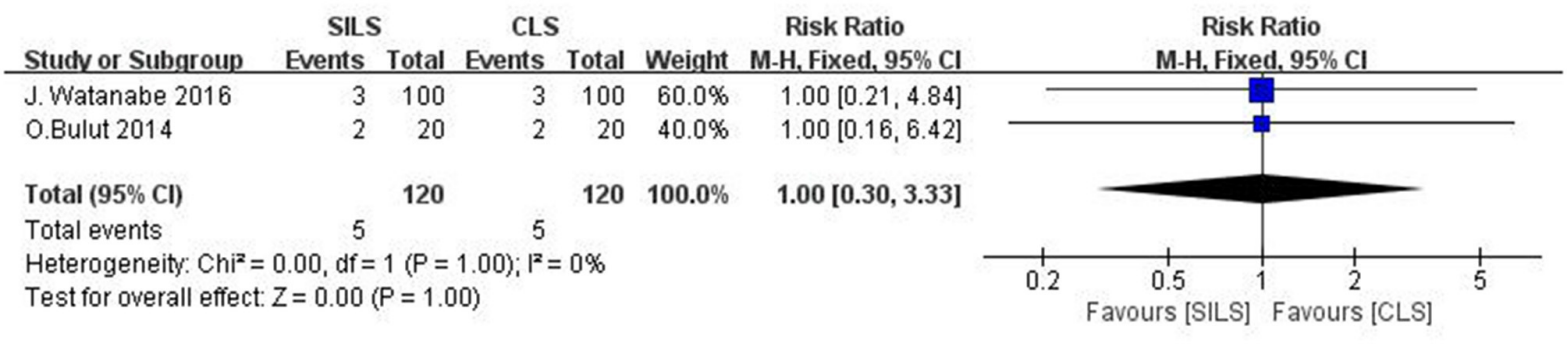
Meta-analysis of reoperation. SILS, single-incision laparoscopic surgery; CLS, conventional laparoscopic surgery.

Two studies (20, 26) with a total of 240 patients reported the rate of reoperation. Heterogeneity test: *P* = 1.00, *I*^2^ = 0, showed no heterogeneity. The fixed-effect model was applied in the analyses. Meta-analysis results [RR = 1.00, 95% CI: 0.30–3.33, *P* = 1.00] showed no statistical significance (Figure 10).

##### Readmission

**Figure 11:**
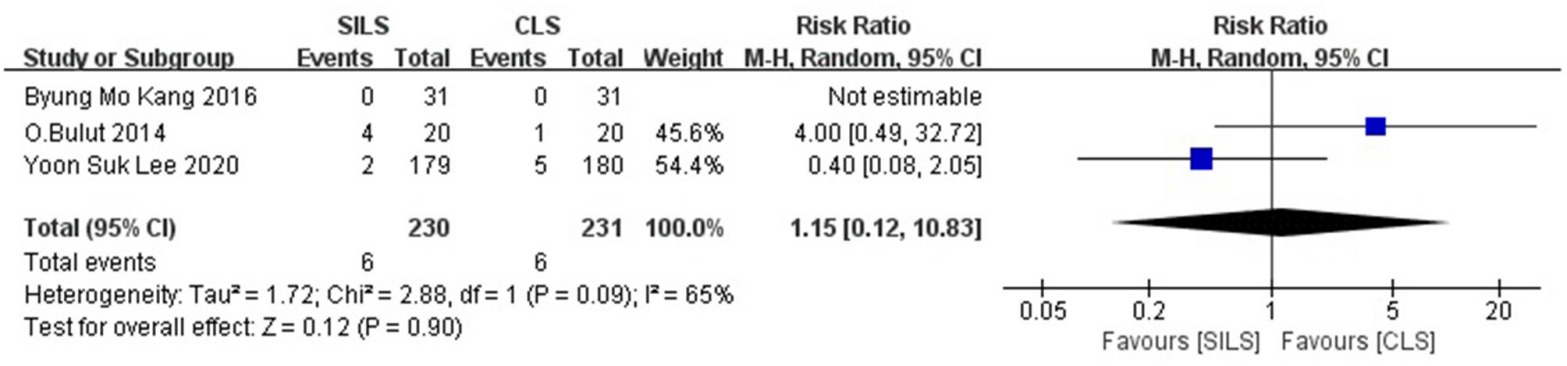
Meta-analysis of readmission. SILS, single-incision laparoscopic surgery; CLS, conventional laparoscopic surgery.

Three studies (20, 22, 23) with a total of 461 patients reported the rate of readmission. Heterogeneity test: *P* = 0.09, *I*^2^ = 65%, showed high heterogeneity. The random-effect model was applied in the analyses. Meta-analysis results [RR =1.15, 95% CI: 0.12–10.38, *P* = 0.90] showed no statistical significance (Figure 11).

#### Publication Bias and Sensitivity Analysis

**Figure 12:**
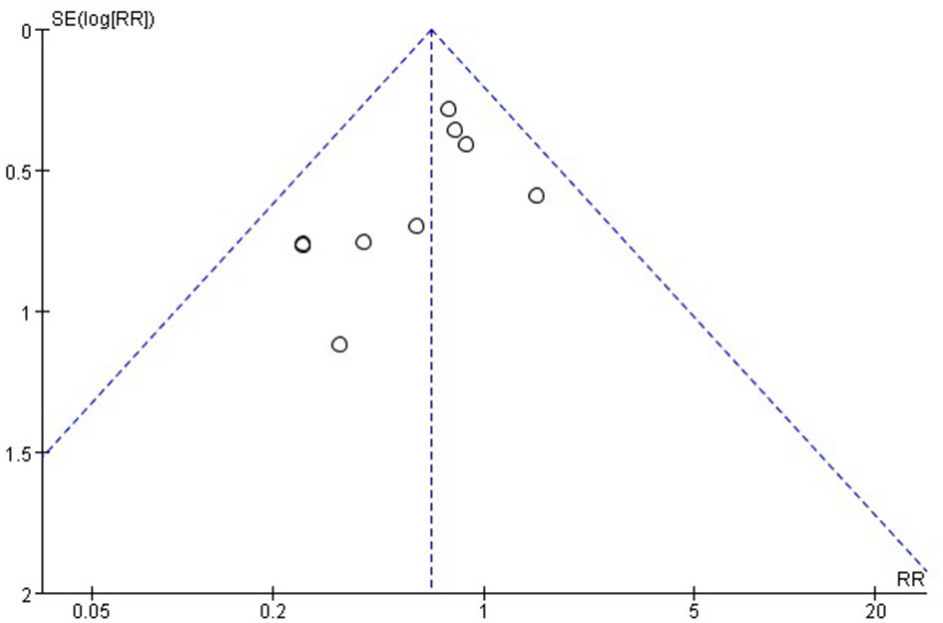
Funnel plot of the studies reporting postoperative complications (20, 21, 24–30).

Publication bias was detected for the main outcomes. An asymmetrical inverted funnel plot for postoperative complications from egger's test (*t* = 1.78, *p* = 0.33), publication bias were not detected as a result of postoperative complications (Figure 12) (20, 28). Publication bias were not detected on intraoperative complications using the egger's test (*t* = 2.41, *p* = 0.14, *p* > 0.05) (21–23). The heterogeneity test for length of abdominal incision showed (*P* = 0.002, *I*^2^ = 76%), high heterogeneity. After excluding a study with low methodological quality (19), there was no observed heterogeneity (*P* = 0.67, *I*^2^ = 0). Therefore, it was concluded that this study was the source of heterogeneity. After deleting the source of heterogeneity, the result of length of abdominal incision using the fixed effects model showed little difference with the previous result, [MD = −1.85, 95%CI: −2.10 to −1.61, *P* < 0.00001]. Heterogeneity test (*P* < 0.00001, *I*^2^ = 89%) of intraoperative blood loss showed high heterogeneity. After excluding two studies with low methodological quality (25, 27), there was no observed heterogeneity (*P* = 0.75, *I*^2^ = 0). Thus, these two studies were considered to be the source of heterogeneity. After deleting the source of heterogeneity, the result of intraoperative blood loss using the fixed effects model showed difference with the previous result, [MD = −15.41, 95% CI: 19.49 to −11.34, *P* < 0.00001]. Heterogeneity test of hospital stay showed high heterogeneity (*P* = 0.0001, *I*^2^ = 80%,). After excluding a study with low methodological quality (27), moderate heterogeneity (*P* = 0.11, *I*^2^ = 46%), was observed. Thus, this study was considered a source of heterogeneity. After deleting the source of heterogeneity, the result of hospital stay using the fixed effects model showed little difference with the previous result, [MD = −0.71, 95% CI: −1.19 to −0.24, *P* = 0.0033]. Sensitive analysis found that the exclusion of any single study did not affect the pooled results and heterogeneity in the meta-analysis (Table 3).

**Table 3:** Sensitive analysis.

**References**	**No. of patients**	**SILS**	**CLS**	**RR or MD (95% CI)**	***P*-value**	**I2 (%)**
**Postoperative complications**						
Kang et al. (22)	62	31	31	0.62 [0.45; 0.87]	0.0057	0.0
Chen et al. (25)	86	43	43	0.67 [0.48; 0.93]	0.0175	0.0
Huscher et al. (21)	32	16	16	0.69 [0.49; 0.95]	0.0235	0.0
Watanabe et al. (26)	200	100	100	0.64 [0.45; 0.91]	0.0136	0.0
Poon et al. (24)	50	25	25	0.68 [0.49; 0.94]	0.0193	0.0
Bulut et al. (20)	40	20	20	0.65 [0.46; 0.91]	0.0127	0.0
Wang et al. (27)	120	60	60	0.71 [0.51; 0.99]	0.0436	0.0
Xu (28)	86	43	43	0.71 [0.51; 0.99]	0.0438	0.0
Lee et al. (23)	359	179	180	0.63 [0.42; 0.92]	0.0176	0.0
Pooled estimate	1035	517	518	0.67 [0.49; 0.92]	0.0130	0.0
**Intraoperative complications**						
Kang et al. (22)	62	31	31	1.94 [0.82; 4.59]	0.1297	0.0
Huscher et al. (21)	32	16	16	2.21 [0.95; 5.14]	0.0651	0.0
Lee et al. (23)	359	179	180	5.00 [0.61; 41.30]	0.1352	0.0
Pooled estimate	453	226	227	2.26 [1.00; 5.10]	0.0494	0.0
**Length of abdominal incision**						
Kang et al. (22)	62	31	31	−2.06 [−2.47; −1.65]	<0.0001	80.0
Chen et al. (25)	86	43	43	−2.00 [−2.50; −1.50]	<0.0001	81.2
Wang et al. (27)	120	60	60	−2.03 [−2.54; −1.52]	<0.0001	77.7
Wu et al. (19)	98	49	49	−1.85 [−2.10; −1.61]	<0.0001	0.0
Xu (28)	86	43	43	−2.11 [−2.54; −1.69]	<0.0001	71.0
Pooled estimate	452	226	226	−2.01 [−2.42; −1.61]	<0.0001	76.1
**Intraoperative blood loss**						
Chen et al. (25)	86	43	43	−9.83 [−21.98; 2.31]	0.1124	91.5
Wang et al. (27)	120	60	60	−11.56 [−19.97; −3.14]	0.0071	84.0
Wu et al. (19)	98	49	49	−5.59 [−14.27; 3.09]	0.2069	82.5
Xu (28)	86	43	43	−5.88[−15.63; 3.88]	0.2375	91.3
Pooled estimate	390	195	195	−8.23 [−16.75; 0.29]	0.0583	88.9
**Number of lymph nodes removed**						
Kang et al. (22)	62	31	31	−0.21 [−0.83; 0.41]	0.5008	0.0
Chen et al. (25)	86	43	43	−0.19 [−0.82; 0.44]	0.5507	26.6
Huscher et al. (21)	32	16	16	−0.23 [−0.86; 0.40]	0.4705	6.3
Wang et al. (27)	120	60	60	−0.19 [−0.83; 0.45]	0.5624	27.3
Wu et al. (19)	98	49	49	−0.46 [−1.31; 0.38]	0.2844	12.6
Xu (28)	86	43	43	0.30 [−0.49; 1.09]	0.4632	0.0
Pooled estimate	484	242	242	−0.17 [−0.79; 0.45]	0.5845	9.8
**Conversion to laparotomy**						
Kang et al. (22)	62	31	31	1.00 [0.33; 3.07]	0.9974	0.0
Watanabe et al. (26)	200	100	100	1.67 [0.53; 5.30]	0.3849	12.6
Bulut et al. (20)	40	20	20	1.60 [0.53; 4.85]	0.4040	10.8
Wu et al. (19)	98	49	49	1.67 [0.52; 5.31]	0.3857	12.2
Lee et al. (23)	359	179	180	0.83 [0.26; 2.69]	0.7605	0.0
Pooled estimate	759	379	380	1.31 [0.48; 3.60]	0.6011	0.1
**Operation time**						
Kang et al. (22)	62	31	31	12.76 [5.89; 19.64]	0.0003	0.0
Chen et al. (25)	86	43	43	11.83 [4.49; 19.16]	0.0016	0.0
Huscher et al. (21)	32	16	16	11.71 [5.08; 18.34]	0.0005	0.0
Wang et al. (27)	120	60	60	11.84 [4.93; 18.75]	0.0008	0.0
Wu et al. (19)	98	49	49	10.21 [2.62; 17.80]	0.0084	0.0
Xu (28)	86	43	43	13.01 [5.22; 20.79]	0.0011	0.0
Pooled estimate	484	242	242	11.90 [5.37; 18.43]	0.0004	0.0
**Hospital stay**						
Kang et al. (22)	62	31	31	−1.40 [−2.17; −0.63]	0.0003	74.5
Chen et al. (25)	86	43	43	−1.14 [−2.06; −0.22]	0.0155	83.9
Huscher et al. (21)	32	16	16	−1.14 [−1.99; −0.29]	0.0088	84.0
Wang et al. (27)	120	60	60	−0.71 [−1.19; −0.24]	0.0033	46.2
Wu et al. (19)	98	49	49	−1.21 [−2.29; −0.13]	0.0281	84.0
Xu (28)	86	43	43	−1.23 [−2.33; −0.13]	0.0281	84.0
Pooled estimate	484	242	242	−1.12 [−1.89; −0.34]	0.0048	80.0
**Reoperation**						
Watanabe et al. (26)	200	100	100	1.00 [0.16; 6.42]	1.0000	0.0
Bulut et al. (20)	40	20	20	1.00 [0.21; 4.84]	1.0000	0.0
Pooled estimate	240	120	120	1.00 [0.30; 3.33]	1.0000	0.0
**Readmission**						
Kang et al. (22)	62	31	31	1.15 [0.12; 10.83]	0.9044	65.2
Bulut et al. (20)	40	20	20	0.40 [0.08; 2.05]	0.2725	/
Lee et al. (23)	359	179	180	4.00 [0.49; 32.72]	0.1961	/
Pooled estimate	461	230	231	1.15 [0.12; 10.83]	0.9044	65.2

*SILS, single incision laparoscopic surgery; CLS, conventional laparoscopic surgery; RR, relative risk; MD, mean difference; 95%CI, 95% confidence interval*.

#### Assessment of the Quality of Evidence

A total of 10 outcome measures were evaluated. Risk bias: Jadad's score of the included studies was ≥4, and were considered high-quality studies. Thus, all outcomes had no risk of bias. However, studies without allocation concealment were considered to have a serious risk of bias. Inconsistency: Due to the high heterogeneity, outcomes of length of abdominal incision, intraoperative blood loss, and length of hospital stay were considered to have serious inconsistencies. Indirectness: All studies were direct comparisons, so indirectness was not significant. Imprecision: The sample size was large enough for outcomes of postoperative complications, conversion to laparotomy, and readmission, thus no imprecision was considered. Other outcomes were assessed serious imprecision due to their small sample size. Publication bias: Evidence of publication bias was detected in the outcome of postoperative complications. Overview: The quality of evidence of the length of abdominal incision, intraoperative blood loss, and length of hospital stay was low. The quality of evidence of intraoperative complications, postoperative complications, number of lymph nodes removed, operation time, and reoperation was moderate. The quality of evidence of conversion to laparotomy and readmission was high (Table 4).

**Table 4:** GRADE evidence profile of outcomes.

**Outcome**	**Number of studies**	**Assessment of evidence quality**					**Number of participants**	**Effect (95%CI)**	**Evidence quality**
		**Risk bias**	**Inconsistency**	**Indirectness**	**Imprecision**	**Publication bias**			
Postoperative complications	9	No	No	No	No	Undetected	1,035	RR = 0.67 (0.49, 0.92)	High
Intraoperative complications	3	No	No	No	Serious	Undetected	453	RR = 2.26 (1.00, 5.10)	Moderate
Length of abdominal incision	5	No	Serious	No	Serious	Undetected	452	MD = −2.01 (−2.42, −1.29)	Low
Intraoperative blood loss	4	No	Serious	No	Serious	Undetected	390	MD = −8.23 (−16.75, 0.29)	Low
Number of lymph Nodes removed	6	No	No	No	Serious	Undetected	484	MD = −0.17 (−0.79, 0.45)	Moderate
Conversion to laparotomy	5	No	No	No	No	Undetected	759	RR = 1.31 (0.48, 3.60)	High
Operation time	6	No	No	No	Serious	Undetected	484	MD = 11.9 (5.37, 18.43)	Moderate
Hospital stay	6	No	Serious	No	Serious	Undetected	484	MD = −1.12 (−1.89, −0.34)	Low
Reoperation	2	No	No	No	Serious	Undetected	240	RR = 1 (0.3, 3.33)	Moderate
Readmission	3	No	Serious	No	Serious	Undetected	461	RR = 1.15 (0.12, 10.83)	Low

## Discussion

SILS, as an emerging minimally invasive technique, attracts a lot of attention from patients and surgeons, because of its potential advantages such as smaller incision length, lower rate of intraoperative complications, and so on. After analyzing several clinical controlled trials, the European Association of Endoscopic Surgery (EAES) pointed out that SILS also has the advantages of better aesthetics and reduced postoperative pain (29). Although high-quality visualization has brought many benefits, single-incision laparoscopic surgery has some weaknesses. Poor ergonomics and technical difficulty are the most important reasons why this technology has not been rapidly adopted. Some scholars (30) believe that SILS has no obvious advantages over CLS, the operation time is longer, and the difficulty in the operation is greatly increased. Therefore, this study analyzed the efficacy of SILS and CLS in the treatment of colorectal cancer based on randomized controlled trials.

A total of 10 RCTs with 1,133 participants were included in this study. No significant difference was found in the mortality of 30 days between SILS and CLS. The meta-analysis results showed that SILS could reduce postoperative complications, length of abdominal incision, and length of hospital stay compared with CLS. However, SILS had poorer intraoperative complications and operation time compared with CLS. In addition, no significant difference was found in intraoperative blood loss, number of lympg nodes removed, the rate of conversion to laparotomy, the rate of reoperation, the rate of readmission and the rate of anastomotic leakage between the two groups.

This meta-analysis confirmed that SILS reduced the rate of postoperative complications. Besides, we inferred that SILS reduced the length of abdominal incision and number of ports, which may be beneficial to wound care and cause less damage for patients (31). Moreover, some studies show that patients who undergo single incision laparoscopic surgery have lower levels of postoperative inflammation than patients who undergo conventional laparoscopic surgery (19, 28). This could be one of the reasons why fewer postoperative complications were reported in the SILS group. The length of abdominal incision in SILS is 2.01 cm shorter than CLS. Besides, SILS not only plays a cosmetic role but also makes the patients think that they are doing a “minor surgery”, which is important for their postoperative mood adjustment. The postoperative recovery time depends on several factors including age, nutritional status, underlying disease, and scope of resection. SILS does not reduce the scope of resection compared with CLS, and apart from the aesthetic advantage, avoiding some small incisions may not affect the speed of recovery. In this meta-analysis, six studies provided data on the length of hospital stay. SILS's length of hospital stay was 1.12 days shorter compared with CLS. However, since the included studies did not use the same discharge standards, the difference in hospital stay has a low reference value. Moreover, the reduction in the length of hospital stay by 1.12 days may not have any clinical significance. This study confirmed that the SILS group had worse rates of intraoperative complications and operation time, compared with the CLS group. These may have been caused by several reasons. First, different levels of experience among surgeons may affect the operation time and rate of intraoperative complications. U-Syn Ha et al. found that the surgical skills acquired by traditional laparoscopic surgeons cannot be directly converted into SILS skills and that novices with laparoscopic surgery can obtain SILS skills similar to those of experienced surgeons through training (32). Another study found that in the absence of practice, SILS skills acquired at 8 weeks deteriorated, while conventional laparoscopic skills were well maintained during the entire 12-week observation period (33). This means that the maintenance of SILS skills differs from conventional laparoscopic surgery, and the maintenance of SILS is more difficult. Second, different specifications of surgical instruments, and inconsistent colorectal cancer surgical methods (such as low anterior resection of rectal cancer, radical resection of abdominal perineum combined with rectal cancer) may also affect the operation time and rate of intraoperative complications. Third, compared with CLS, SILS is an emerging technology, and surgeons require a certain degree of operation proficiency. SILS requires direct insertion of the operating instruments into the abdominal cavity through a single incision in the abdominal wall in a nearly parallel manner. Operating under the limited surgical view, lack of effective traction, equipment crowding, and collision during the operation, make SILS more difficult, resulting in prolonged operation time and increase the rate of intraoperative complications (34).

The rate of conversion to laparotomy is an outcome that surgeons may be interested in. For SILS surgery, there is a transition option: conversion to CLS, but for CLS, it can only be directly converted to open surgery, which makes it meaningless to compare the rate conversion to CLS between the two groups. In the studies we included, the definitions of conversion to CLS cannot be unified. Conversion to CLS was defined as the insertion of additional trocars during SILS in two studies (20, 22), but in other studies, conversion to CLS was defined as the addition of two or more trocars (23, 26). The definition of conversion to laparotomy was that a skin incision longer than designated incision was required to extract the resected specimen or to control intraoperative complications in two studies (20, 22), but in another study, conversion to laparotomy was defined by a wound length measuring 8 cm or greater (26). We believe that the definition of conversion to laparotomy between different studies has low clinical heterogeneity. Although the meta-analysis did not show a statistical difference between the two groups, the subgroup analysis suggested that in colon cancer patients, the rate conversion to open surgery of SILS was higher than that of CLS, and the data was consistent.

The long-term outcome from the SIMPLE study showed that SILS did not have an absolute advantage (23, 35). Although there were some statistical differences in the overall quality of life scores, functional scores, and symptom scores at different measurement points after surgery, these statistical differences do not always indicate that SILS has more advantages or disadvantages than CLS. Moreover, these differences can be explained by type I errors caused by multiple hypothesis tests.

To further reduce clinical heterogeneity, we performed subgroup analysis according to the cancer type and previous history of major abdominal surgery. The SILS group showed lower rates of postoperative complications compared with the CLS group in all subgroups. A comparison of the rate of postoperative complications in patients with colorectal cancer, colon cancer, and rectal cancer in the SILS and CLS group, we found that the relative risk (RR) of patients with colon cancer [RR = 0.80, 95% CI: 0.54–1.18, *P* = 0.26] was higher than that of colorectal cancer [RR = 0.61, 95% CI: 0.34–1.07, *P* = 0.09] and rectal cancer patients [RR = 0.54, 95% CI: 0.16–1.89, *P* = 0.34]. We hypothesized that the colon has more blood vessels, which may cause more vascular injury complications and increase the difficulty of surgery. Therefore, SILS for colon cancer can cause postoperative complications, thus increasing the RR of patients with colon cancer. The RR of postoperative complications in patients with major abdominal surgery history [RR = 0.82, 95% CI: 0.50–1.33, *P* = 0.41] was higher than the RR of postoperative complications in patients with no major abdominal surgery history [RR = 0.63, 95% CI: 0.41–0.99, *P* = 0.05]. We hypothesized that patients with major abdominal surgical history have a worse physical condition, and SILS may cause severe damage to these patients, thus, resulting in more postoperative complications. The SILS group showed longer operation time compared with the CLS group in patients with colorectal and colon cancer, and the MD of colorectal cancer patients [MD = 14.28, 95% CI: 5.67–22.9, *P* = 0.001] was higher than the MD of colon cancer [MD = 7.31, 95% CI: −10.89–25.51, *P* = 0.43]. We infer it is determined by the level of surgical skill in different countries. The included studies on colorectal cancer were all from China, while those on colon cancer were from Korea and Italy, which are considered to have a higher level of surgical skills compared with China. Moreover, the two countries have a higher level of training for SILS. Therefore, the operation time of SILS and the MD of operation time in colorectal cancer patients were found to be longer than in the colon cancer group. The colorectal cancer subgroup analysis showed that, the SILS group had a shorter hospital stay than the CLS group [MD = −1.84, 95% CI: −3.30 to −0.38, *P* = 0.01], while colon cancer subgroup analysis showed that the SILS group had a similar length of hospital stay compared with the CLS group [MD = 0.06, 95% CI: −0.69–0.82, *P* = 0.87]. We hypothesized that colon cancer patients have higher rate of postoperative complications, which caused longer hospital stay. The above explanation may also be affected by the instability caused by the reduction in the sample size of the subgroup analysis.

A meta-analysis published by Gu et al. was the closest to our study in terms of structured clinical issues (PICO) (36). Our findings differed from that study in almost all outcomes. We carefully analyzed and speculated that the most likely reason for the difference is that our meta-analysis only included RCTs, and the above meta-analysis also included propensity-score matched studies. The apparently higher heterogeneity (I square) in the above meta-analysis supports our speculation. The randomized controlled trials included in the two meta-analysis are almost the same, and we have reason to believe that the meta-analysis results of the two based on the same randomized controlled trial should also be the same. Future research should focus on comparing data from randomized controlled trials with data from propensity-score matched studies.

Compared with other previous meta-analysis (37–40) including retrospective studies or clinical controlled trials (CCTs), this meta-analysis only included and analyzed all relevant RCTs in the present to ensure that the results were more reliable. However, this study has some limitations. First, the literature included in this study mostly comes from China and Korea, thus, the study results are poorly extrapolated. Secondly, the included literature lacks long-term follow-up results, including the rate of local tumor recurrence or distant metastasis, and survival rate. Thirdly, only two studies blinded the participants, while the others were open-labeled RCTs, which can lead to substantial implementation bias. None of the studies reported whether outcome evaluators were blinded, so measurement bias may also have influenced the results, especially in those subjective outcomes such as length of stay in the hospital. Finally, the sample size of included studies is generally small. Therefore, the above conclusions need to be verified using well-designed long-term large sample RCTs. This systematic review and meta-analysis did not prove that the SILS has a comprehensive and obvious advantage over the CLS. Although SILS for colorectal cancer showed advantages including shorter incision length, lower postoperative complication rates, and shorter hospital stay compared with CLS. Some poor short-term outcomes of SILS, such as longer operation time and more intraoperative complications, suggest that it should be considered carefully. Surgeons should fully discuss the pros and cons of the two surgical procedures with patients, and make a selection based on factors such as the surgeon's experience and training level, surgical facilities, and patient values. RCTs focusing on long-term outcomes are warranted to provide more information on clinical options.

## Data Availability Statement

The datasets presented in this study can be found in online repositories. The names of the repository/repositories and accession number(s) can be found in the article/supplementary material.

## Author Contributions

YY is the principal investigator with overall responsibility for the original draft, together with JJ and HJ wrote the draft and submitted the PROSPERO registration. LD performed searching for relevant studies, data collection, and data analysis. RY, XF, FY, and WL provided help in designing, data analysis, and editing of the manuscript. All authors read and approved the final manuscript.

## Conflict of Interest

The authors declare that the research was conducted in the absence of any commercial or financial relationships that could be construed as a potential conflict of interest.

## Publisher's Note

All claims expressed in this article are solely those of the authors and do not necessarily represent those of their affiliated organizations, or those of the publisher, the editors and the reviewers. Any product that may be evaluated in this article, or claim that may be made by its manufacturer, is not guaranteed or endorsed by the publisher.
